# Toward the Production of Hydroxyapatite/Poly(Ether-Ether-Ketone) (PEEK) Biocomposites: Exploring the Physicochemical, Mechanical, Cytotoxic and Antimicrobial Properties

**DOI:** 10.3390/polym16172520

**Published:** 2024-09-05

**Authors:** Meirilany Rozeno Costa, José Adeilton Carvalho Filho, Carlos Bruno Barreto Luna, Gleydis Manalig Pereira Dantas, Ana Cristina Figueiredo de Melo Costa, Nadja Maria da Silva Oliveira

**Affiliations:** 1 Ceramic Materials Synthesis Laboratory, Federal University of Campina Grande, Av. Aprígio Veloso, 882, Bodocongó, Campina Grande 58429-900, PB, Brazil; adeiltoncarvalho87@yahoo.com.br (J.A.C.F.); manaliggg@gmail.com (G.M.P.D.); ana.figueiredo@professor.ufcg.edu.br (A.C.F.d.M.C.); 2 Polymer Processing Laboratory, Federal University of Campina Grande, Av. Aprígio Veloso, 882, Campina Grande 58429-140, PB, Brazil; brunobarretodemaufcg@hotmail.com; 3 Postgraduate Program in Health Science and Technology—PPGCTS, Dentistry Department, State University of Paraíba, R. Baraúnas, 351, Bodocongó, Campina Grande 58429-500, PB, Brazil; nadjamso@paqtc.org.br

**Keywords:** biomaterials, hydroxyapatite, PEEK, high-energy ball milling, bone repair, mechanical properties, cytotoxicity

## Abstract

The development of hydroxyapatite (HAp) and polyether ether ketone (PEEK) biocomposites has been extensively studied for bone repair applications due to the synergistic properties of the involved materials. In this study, we aimed to develop HAp/PEEK biocomposites using high-energy ball milling, with HAp concentrations (20%, 40%, and 60% *w*/*v*) in PEEK, to evaluate their physicochemical, mechanical, cytotoxicity, and antimicrobial properties for potential applications in Tissue Engineering (TE). The biocomposites were characterized by structure, morphology, apparent porosity, diametral compression strength, cytotoxicity, and antimicrobial activity. The study results demonstrated that the HAp/PEEK biocomposites were successfully synthesized. The C2 biocomposite, containing 40% HAp, stood out due to the optimal distribution of HAp particles in the PEEK matrix, resulting in higher compression strength (246 MPa) and a homogeneous microstructure. It exhibited antimicrobial activity against *Staphylococcus aureus*, *Pseudomonas aeruginosa*, and *Escherichia coli*, with no cytotoxicity observed. These properties make the C2 biocomposite promising for regenerative medicine applications, combining mechanical strength, bioactivity, and biocompatibility.

## 1. Introduction

According to the Global Burden of Diseases, Injuries, and Risk Factors Study, there were 178 million (95% uncertainty interval, 162–196) new fractures globally in 2019, representing a 33.4% increase since 1990, with 455 million prevalent cases of acute or long-term symptoms from a fracture [1]. In Brazil, injuries or other conditions caused by external factors have been among the top five leading causes of hospital admissions. It is important to note that among the morbidities categorized in the “injuries due to external causes” chapter of the eleventh revision of the International Classification of Diseases (ICD-11), bone fractures are the most frequent hospitalization cases, accounting for 43.1% of the total [2].

AO Trauma International and the Orthopaedic Trauma Association (AO/OTA) indicate that the repair process is complex, involving multiple stages that begin with immediate reactions to injury, including damage to soft tissues, blood vessel rupture, and bone necrosis, all co-occurring. The hematoma response triggers the release of inflammatory cells, activates osteoclasts and mesenchymal stem cells, and stimulates chondrocytes to form a rich, soft callus. Cycles of osteoblast and osteoclast activity subsequently restore bone morphology and mechanical properties [3,4,5].

The global orthopedic implants market is expected to reach over $64 billion by 2025, driven by factors such as an aging population, increased sports injuries, and the growth of minimally invasive surgeries [6]. Although orthopedic implants made from metals, ceramics, and synthetic polymers have been successful, each material class has limitations that impact long-term performance [7]. Metals are rigid and durable but can corrode and cause adverse tissue reactions over time [8]. Ceramics have high compressive strength but are brittle and prone to fractures. On the other hand, polymers better match the rigidity of bone but lack robustness and bioactivity [9]. This has driven the research into new biomaterials that can overcome the deficiencies of current implants. Choosing an implant to restore structural integrity is crucial for treatment, as it can avoid trauma to the donor site, reduce patient pain, and lower the overall treatment cost, providing a new strategy for the clinical repair of bone defects [10,11].

Bone substitutes should be biocompatible, bioresorbable, osteoconductive, osteointegrative, non-immunogenic, and non-inflammatory. Additionally, various technological strategies can be applied for the “customization” of treatment and effectiveness in bone regeneration, as these materials can enhance the irregularity of tissue defects and reduce inflammation and infection in the application area [12,13,14,15]. For example, a study conducted by Safavi et al. [16] evaluated the osteogenic activity of NiTi orthopedic implants through HAp-Nb_2_O_5_. composite coatings. The results demonstrated that the incorporation of HAp-Nb_2_O_5_ reinforcement particles leads to a significant increase in adhesion, proliferation, and osteogenic activity of SAOS-2 cells (osteosarcoma). In this context, hydroxyapatite (HAp) (Ca_10_(PO_4_)_6_ (OH)_2_) has such characteristics, and additionally, its biological application is due to its chemical similarity to the inorganic components of human bones and teeth, being the main component in the form of calcium apatite [14]. This bioceramic calcium phosphate can be obtained from natural and synthetic sources and is hydrophilic and stable even at high temperatures [17]. However, it has some disadvantages when used in isolation, as it is highly brittle, with low tensile strength (18 MPa) and high compressive strength (917 MPa), making it insufficient to promote vascularization and osteoinductivity [18].

Thus, combining bioactive inorganic phases with polymers can produce composites with tailored biological and mechanical properties [19]. For example, in a study conducted by Ma et al. [20] using a composition and injection molding technique, they assessed the bioactivity of a polyether ether ketone (PEEK) biocomposite with HAp. The in vitro bioactivity evaluation demonstrated cellular attachment and proliferation of MC3T3-E1 cells (pre-osteoblast cell type) on the material’s surface, showing enhanced spreading efficiency. Additionally, this composite induced the formation of an apatite layer, and the attracted osteoblasts proliferated and differentiated to produce collagen and protein, which mineralized to form a new bone interface. Another study conducted by Qiu et al. [21] examined the osteointegration and antimicrobial properties of PEEK implants by applying a double-layer coating of amorphous magnesium phosphate (AMP) and HAp, where the PEEK was sulfonated using an ultrasonic method and the AMP/HAp was processed using microwave technology. The results demonstrated that surface modification conferred antibacterial properties, highlighting the potential of these composite coatings in preventing infections.

It is important to note that PEEK has been approved by the U.S. Food and Drug Administration (FDA) as an implant material since the 1980s [22]. It belongs to a family of high-temperature thermoplastic polymers, semicrystalline with chemical stability and an elastic modulus around 3.6 GPa, close to bone tissue [23]. Despite its high macromolecular rigidity, its planar conformation allows the polymer chains to be organized into crystalline and amorphous domains [24]. Biocomposites made from this polymer or surface modifications have been prepared using various methodologies such as direct injection molding, three-dimensional (3D) printing technology [25], sol–gel processing [26], plasma spraying [27,28], and physical or chemical vapor deposition [21]. However, they have limitations such as multi-step preparation processes and high costs. Additionally, the high cost of implant-grade raw materials, such as PEEK, can lead companies to face large-scale process reproducibility challenges. Therefore, alternative methods to achieve higher productivity in the production of these implants should be considered, as the level of crystallinity and crystalline morphology can influence the physical properties of PEEK-containing composites [29].

Thus, high-energy ball milling can ensure optimized processing through a single-step methodology, such as physical mixing, allowing for high reproducibility. Moreover, it can lead to a more uniform distribution of HAp particles within the PEEK matrix, and this homogeneity is essential for achieving consistent mechanical and biological properties throughout the material. Therefore, this study aimed to evaluate the properties of HAp/PEEK biocomposites formed by high-energy milling.

## 2. Materials and Methods

### 2.1. Materials

The biocomposites, a significant advancement in materials science, were produced using the following materials: Victrex^®^ polyether ether ketone (PEEK) under the commercial code Vicote 702. Hydroxyapatite (HAp) was synthesized by precipitation at the Laboratory of Ceramic Materials Synthesis (LabSMaC, Campina Grande, Brazil), and acetone was 99%.

### 2.2. Synthesis of Biocomposites by High-Energy Ball Milling

The synthesis of HAp followed the methodology proposed by Saeri et al.; Barandehfard et al.; and Sarkar et al. [30,31,32], using the precipitation method with a phosphorus/calcium ratio of 1.67. Initially, solutions of calcium hydroxide (Ca(OH)₂) and phosphoric acid (H₃PO₄) were prepared at 2 M. The calcium hydroxide solution was subjected to constant stirring for 30 min in an IKA^®^ RH essential KT/C mixer/heater until it reached a temperature of 80 °C. After this, the H₃PO₄ solution was added dropwise while stirring at 100 rpm. The solution was left in an oven at 110 °C for 24 h. The product was sifted through an ABNT 100 mesh sieve (150 µm). The produced material was then characterized and used to prepare the biocomposite formulations.

**Figure 1:**
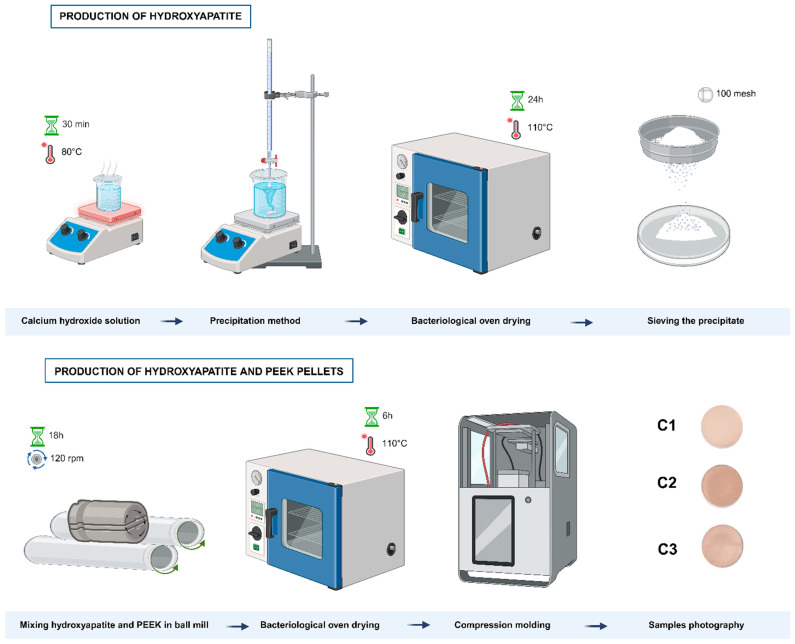
Manufacturing process of the biocomposites.

The addition of HAp to PEEK, based on the mass/volume ratio, was carried out in proportions of 20% (C1), 40% (C2), and 60% (C3). The proportions of HAp were determined based on bone composition, which has approximately 70% inorganic phase [33]. A ball mill was used to mill a PEEK/HAp composite using alumina balls as the grinding medium. The ratio between the weight of the grinding balls and the weight of the material was optimized to maximize milling efficiency and ensure a homogeneous material distribution. A jar with a total volume of 225 mL was used, and alumina balls with a diameter of 5 mm were added. The total mass of the balls used was 270 g. In addition to the alumina balls, 23.33 g of PEEK/HAp composite powder was added to the jar. This configuration provides enough space for the balls to move during the milling process, promoting effective impacts and adequate milling of the PEEK/HAp powder. The milling time was set to 18 h, allowing the material to be sufficiently processed and ensuring uniformity without causing excessive milling. The rotation speed was adjusted to 120 rpm, which is ideal for avoiding material segregation and ensuring efficient mixing. Figure 1 shows the production flowchart of PEEK/HAp biocomposites.

### 2.3. Characterization

#### 2.3.1. X-ray Diffraction (XRD)

The phases present and crystallinity were determined from X-ray diffraction (XRD) data using a BRUKER diffractometer (Bruker, Billerica, MA, USA), model D2 Phaser, with copper radiation (CuKα1 = 1.54056 Å) at 40 kV and 30 mA. The scanning was performed in the range of 10° ≤ 2θ ≤ 80°, with a speed of 0.016°/min and a time of 5 s.

#### 2.3.2. Fourier Transform Infrared Spectroscopy (FTIR)

The spectra obtained in the Fourier Transform Infrared (FTIR) region were recorded using a BRUKER VERTEX 70 FT-IR (Bruker, Billerica, MA, USA), model 660-IR, between 4000 and 500 cm^−1^, with a resolution of 4 cm^−1^ and 32 scans.

#### 2.3.3. Scanning Electron Microscopy (SEM/EDS)

The morphology and the presence of clusters were analyzed using Scanning Electron Microscopy (SEM) with a VEGA 4, TESCAN (TESCAN, Brun, Czech Republic), operating between 10 and 15 mA, using a magnification of 5000×. The samples were gold-coated.

#### 2.3.4. Apparent Porosity Determination

For the apparent porosity (PA) test, samples with a thickness of 3.94 mm and a diameter of 22.6 mm were used. The evident porosity test was based on Archimedes’ principle. The samples were prepared and dried at 345 °C for 30 min to obtain the dry weight (Ps). Subsequently, they were immersed in 20 mL of distilled water for 24 h, and the immersed weight (Pi) and the wet weight (Pu) were measured.

#### 2.3.5. Compressive Strength Mechanical Testing

This was performed using an INSTRON 5582 universal testing machine (INSTRON, São Paulo, Brazil), with a load of 10 N and a speed of 1.3 mm/min (ASTM D 695-23) [34], with one grip fixed and the other mobile. Three samples were tested for each batch, with tests conducted at room temperature between 20 and 25 °C. The average values and standard deviations were calculated based on the three samples from each group.

#### 2.3.6. Cytotoxicity Testing

Cytotoxicity was assessed using the Agar Diffusion method, according to ISO 10993-5:2009 [35], and adapted by Pina et al. and Wanderley et al. [36,37]. This method assesses the effects of the material on cells by using an agar layer that allows the diffusion of chemical substances from the sample to the cell layer [38,39]. The L929 fibroblast cell line (ATCC NCTC clone 929, Rio de Janeiro Cell Bank, Rio de Janeiro, Brazil) was cultured in RPMI 1640 medium (Gibco—Invitrogen Corporation, Grand Island, NE, USA) and incubated in a humidified incubator at 37 °C and 5% CO_2_ until 80% confluence was reached. Subsequently, trypsinization with 0.25% trypsin (Gibco^®^, Life Technologies, Carlsbad, CA, USA) and cell counting was performed using an automated cell counter Interwoven—Thermo Fisher (Waltham, MA, USA). Suspensions of 1.0 × 10^5^ cells/mL were distributed into 6-well plates, with 4 mL added to each well, and incubated under the same conditions described above for 24 h. Upon reaching uniform 80% confluence in the plates, a detailed examination was conducted using a microscope, the culture medium was aspirated, and 1 mL/well of medium prepared with MEM 2X concentrated (Gibco^®^—Invitrogen Corporation, Grand Island, NE, USA) and 1.8% agar solution with 0.01% neutral red (Sigma–Aldrich, St. Louis, MI, USA) was added. This was kept in the dark at room temperature for 10 minutes to solidify. Test samples of 1 cm^2^ were placed in the center of the agar surfaces, along with positive (latex sheet) and negative (high-density polyethylene—HDPE) controls in triplicate. The plates were wrapped in aluminum foil and incubated upside down for 24 h in a humid chamber at 37 °C and 5% CO_2_. In the end, following the guidelines of ISO 10993-5:2009, the areas of discoloration around the sample were measured, and an inverted microscope Nikon Eclipse TS100 (Minato, Tokyo, Japan) was used to analyze cell lysis, with images recorded after 24 h of incubation. The discolored areas and lysis were graded according to the ISO 10993-5:2009 guidelines, as shown in Table 1, as follows: 0 = no detectable cell lysis; 1 = less than 20% cell lysis; 2 = 20–40% cell lysis; 3 = 40–60% cell lysis; 4 = 60–80% cell lysis; 5 = more than 80% cell lysis.

#### 2.3.7. Determination of Antimicrobial Activity from the Minimum Inhibitory Concentration (MIC)

##### Preparation of Bacterial Suspension and Inoculum Standardization

To determine antimicrobial activity based on the minimum inhibitory concentration (MIC), microbial strains from the American Type Culture Collection (ATCC) were used: *S. aureus* (ATCC 25923), *P. aeruginosa* (ATCC 27853), and *E. coli* (ATCC 25922). These strains were maintained at the Drug Development and Testing Laboratory of UEPB (Labdem-UEPB), stored in brain–heart infusion broth (BHIB) (DIFCO^®^) and 20% (*v/v*) glycerol. The inoculum was standardized according to Clinical Laboratory Standards Institute (CLSI) M07 guidelines, using a Mueller Hinton broth (MHB) (DIFCO^®^) culture incubated for 24 h at 35 ± 2 °C. The inoculum was standardized to achieve a concentration corresponding to a MacFarland 0.5 scale in MHB, and for the assays, the initial inocula were diluted to a concentration of 2.0 to 8.0 × 10^5^ CFU/mL [40].

##### Broth Microdilution Method for Minimum Inhibitory Concentration Determination

The minimum inhibitory concentrations (MICs) were determined in 96-well microdilution plates following the methodologies outlined by the Clinical and Laboratory Standards Institute M07 and M27 (CLSI, 2018). To this end, 190 μL of Mueller Hinton broth was added to each well of sterile round-bottom plates, and then 10% (*w*/*v*) of the PEEK/HAp C2 powder from the test compounds was added. Serial dilutions of the composite were performed to obtain final concentrations ranging from 100 × 10^3^ µg/mL to 390.62 µg/mL. Each well received 10 μL of a bacterial suspension with a final concentration of 1.5 × 10^8^ CFU/mL. After treatment, the plates were incubated at 35 ± 2 °C for 24 h. The positive and negative controls involved bacterial suspension combined with broth and only the culture medium. Antibiotics such as ciprofloxacin hydrochloride (CIPRO), oxacillin (OXA), and ceftazidime (CFT) were used as positive controls at a concentration of 200 µg/mL. After the incubation period, 20 μL of 2% triphenyl tetrazolium chloride (TTC) in sterilized water was added, and after 1 hour of incubation, visual readings were performed.

## 3. Results and Discussion

**Figure 2:**
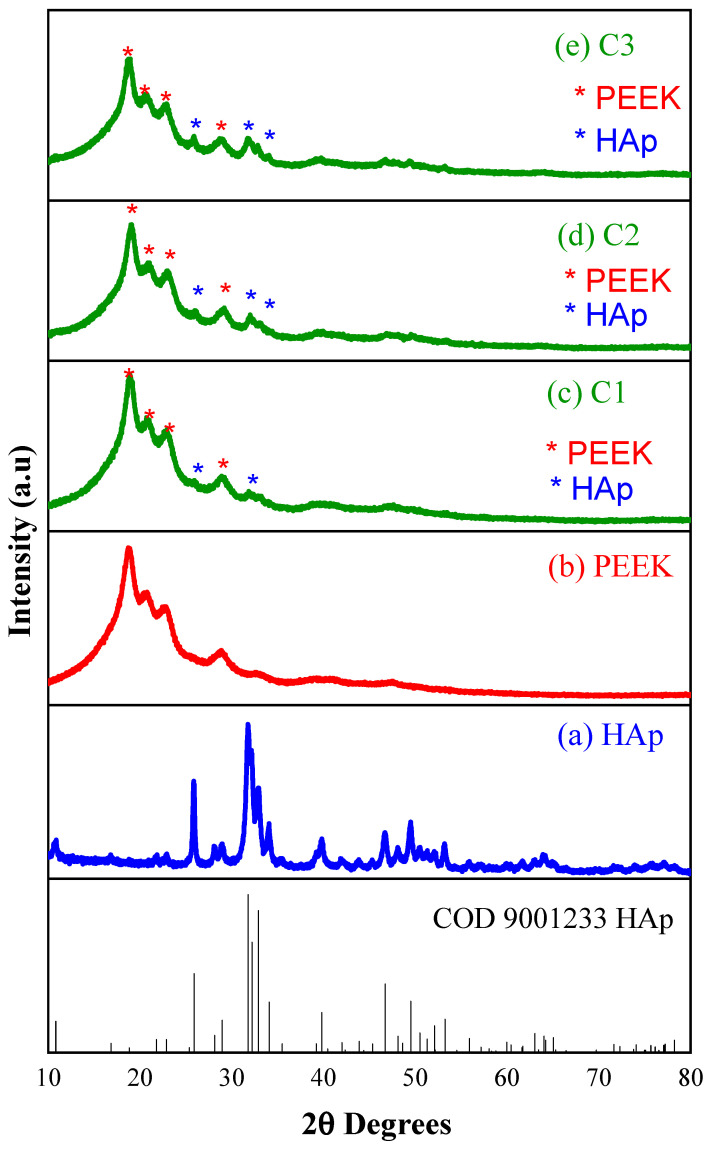
X-ray diffractograms: (**a**) HAp, (**b**) PEEK, (**c**) C1, (**d**) C2, and (**e**) C3.

Figure 2 presents the X-ray diffractograms of HAp, PEEK, and the composites with varying HAp contents. In Figure 2, it can be observed that calcium HAp was successfully obtained using the precipitation method, as evidenced by the presence of characteristic peaks corresponding to the crystal planes (2 1 3), (2 2 2), (3 1 0), (2 0 2), (1 1 2), (2 1 1), (2 1 0), and (0 0 4) at approximately 26°, 31.8°, 33°, 40°, 46°, and 50°, respectively. This behavior was also reported by Li et al. [41], who studied the 3D printing of PEEK biocomposites with Ca_10_(OH)(PO_4_)_3_ for bone implants. For PEEK, as shown in Figure 2b, a broadband with four peaks corresponding to the diffraction planes (110), (111), (200), and (211) was observed at around 18.8°, 21°, 22.6°, and 28.7°, respectively. This is typical of a semicrystalline polymer. These characteristics were also reported by Asante et al. [42], who studied sulfonated carbon fiber-reinforced PEEK at room temperature with HAp coating.

The diffractograms in Figure 2c–e show that the biocomposites were formed by adding 20%, 40%, and 60% HAp to PEEK, respectively. It can be noted that the addition of HAp did not significantly alter the characteristics of the PEEK matrix, as the main diffraction peaks characteristic of both HAp and PEEK are displayed. This behavior demonstrates that the HAp/PEEK biocomposite was successfully synthesized. Such characteristics were also reported by Ma et al., Qi et al., and Yusong et al. [20,43,44], when evaluating the bioactivity of a HAp-incorporated PEEK biocomposite.

**Figure 3:**
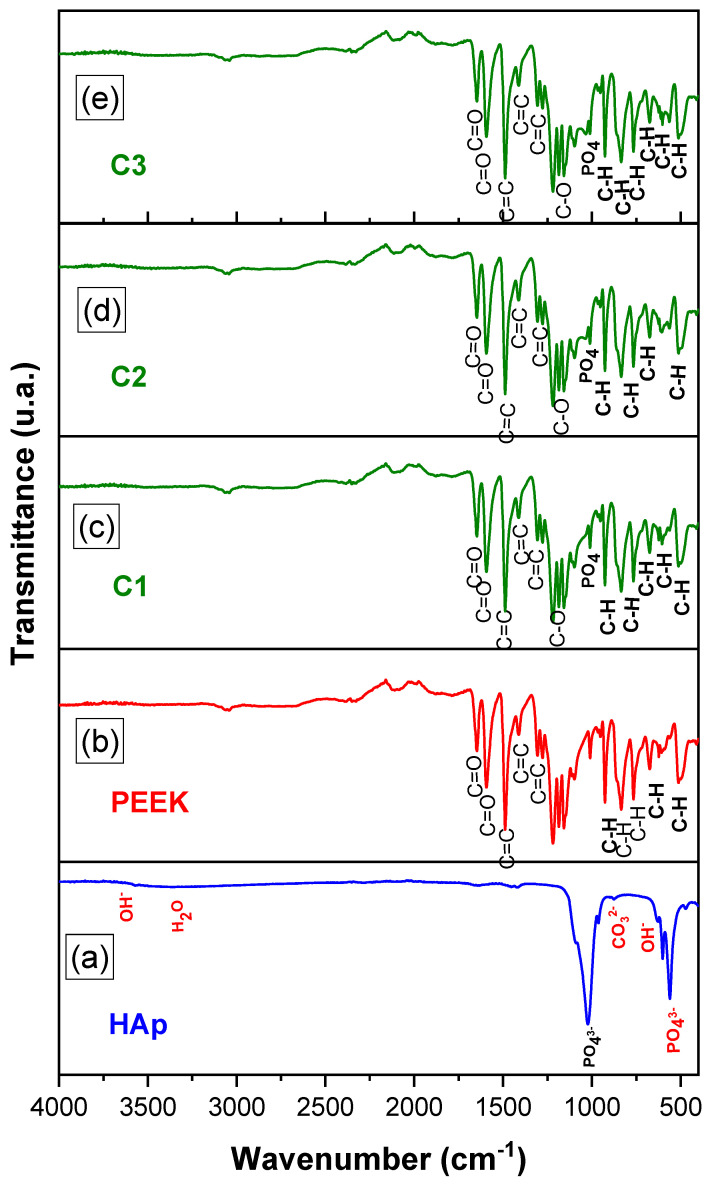
Infrared spectra: (**a**) HAp, (**b**) PEEK, (**c**) C1, (**d**) C2, and (**e**) C3.

Figure 3 illustrates the FTIR spectra of HAp, PEEK, and the biocomposites, as a function of HAp content, in the range of 4000–500 cm^−1^.

When analyzing the FTIR spectrum of HAp, characteristic bands for HAp functional groups are observed around 1026 cm^−1^ and 1094 cm^−1^, corresponding to the asymmetric stretching vibration of the PO_4_^3−^ group, and another at 960 cm^−1^, associated to the symmetric stretching vibrations of PO_4_^3−^. A band at 871 cm^−1^ is attributed to the stretching vibrations of CO_3_^2−^, indicating the presence of carbonate ions in phosphate units (type B substitution). Absorption bands appearing at 3569.59 cm^−1^and 632.53 cm^−1^ correspond to the stretching and bending vibrations of structural OH. These characteristic absorption bands of HAp are consistent with results obtained by Xu et al., Nascimento et al., and Tabrizi et al. [45,46,47].

The FTIR spectrum of PEEK showed absorption bands for aromatic CH groups at 671, 766, 830, and 929 cm^−1^, suggesting angular deformation of the aromatic CH group. Bands at 1011, 1159, 1170, and 1213 cm^−1^ correspond to asymmetric stretching of the C-O group. Axial deformation bands at 1278, 1305, and 1488 cm^−1^ are associated with the C=C group, related to the aromatic ether C-O group. Bands at 1594 and 1645 cm^−1^ correspond to the primary and secondary stretching of the carbonyl C=O group, respectively. These bands were also reported by [48], who studied the characterization of the chemo-mechanical properties of polyether ether ketone (PEEK).

From the FTIR spectra of the HAp/PEEK biocomposites (Figure 3c–e), a band at 1000 cm^−1^ was observed, corresponding to the asymmetric stretching deformation of the PO_4_^3^ group. The bands at 671, 766, 830, and 929 cm^−1^ are associated with PEEK, suggesting angular deformation of the aromatic CH group. Bands at 1011, 1159, 1170, and 1213 cm^−1^ indicate asymmetric stretching of the C-O group. Bands at 1594 and 1645 cm^−1^ correspond to the primary and secondary stretching of the carbonyl C=O group, respectively. The results indicate that HAp is incorporated into the PEEK chain, which is essential for enhancing the functional behavior of the biocomposite.

**Figure 4:**
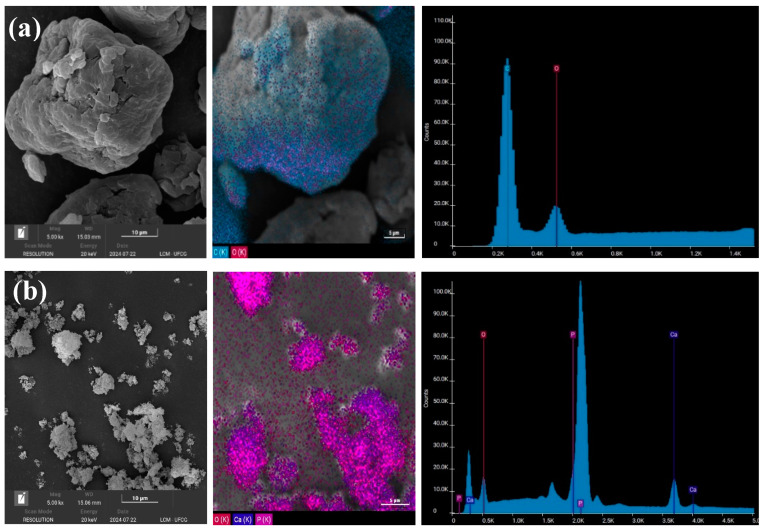

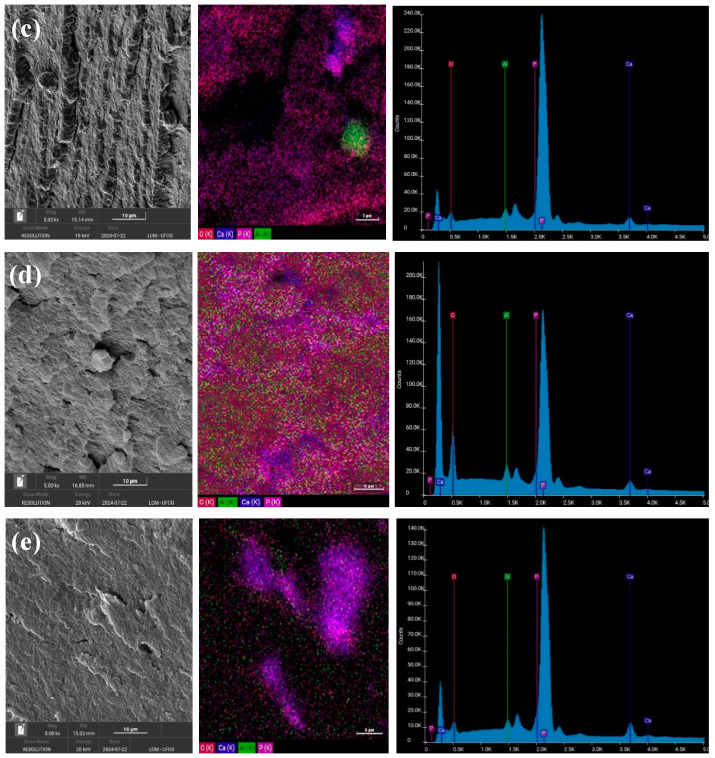
SEM images with chemical mapping by EDS, for: (**a**) HAp, (**b**) PEEK, (**c**) C1, (**d**) C2, and (**e**) C3.

Figure 4 shows the scanning electron microscopy (SEM) micrographs, EDS spectra, and EDS mapping of HAp, PEEK, and the biocomposites, at a magnification of 5000×. Figure 4a demonstrates that the HAp sample consists of irregular, rounded aggregates with a broad distribution. This morphology is typical of HAp synthesized by coprecipitation, as also reported by Afshar et al. [49]. According to Ponciano et al. [50], this synthesis route produces nanometric particles that enhance bone tissue bonding, cell proliferation, and better dispersion. The EDS spectrum confirmed the predominant presence of calcium and phosphate, the main elements in the chemical composition of HAp.

In Figure 4b, it can be seen that PEEK exhibits a morphology of rounded grains with a dense and spherical structure, characteristic of PEEK, as also reported by Manzoor et al. [51]. EDS mapping showed a homogeneous distribution of carbon and oxygen elements, while the EDS spectrum highlighted the prominent peaks of these two elements, confirming the predominant chemical composition of PEEK.

Regarding the HAp/PEEK biocomposites, the presence of distributed granules was observed, indicating that the increased amount of HAp favored more significant densification and interaction of the HAp particles, which tend to form agglomerates due to Van der Waals forces and hydrogen bonding [52]. In Figure 4e, the HAp particles are distributed heterogeneously on the surface, with a discrete increase in particle agglomerates of various sizes and shapes dispersed on the PEEK surface. In Figure 4d, the HAp particles are dispersed uniformly within the PEEK matrix. The addition of HAp makes the PEEK surface more hydrophilic, which can facilitate cell adhesion to the surface of an implant and, consequently, promote bone growth [20].

**Figure 5:**
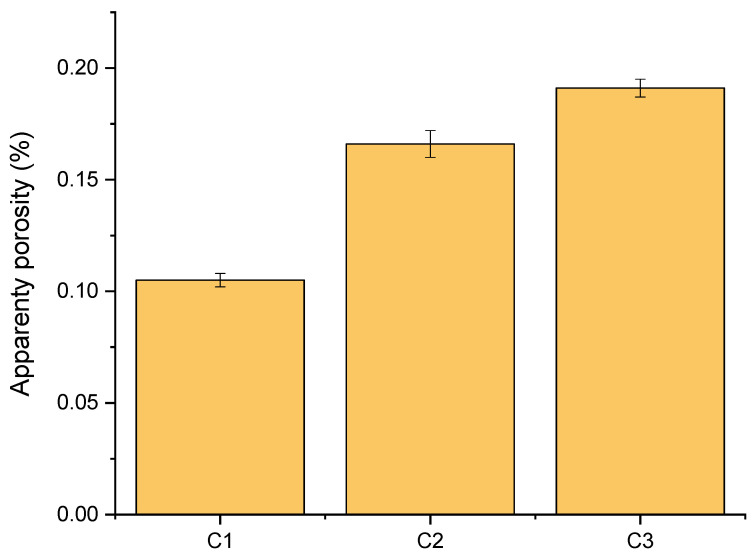
Histogram of apparent porosity of the C1, C2, and C3 biocomposites.

Figure 5 illustrates the apparent porosity of the HAp/PEEK biocomposites with different HAp concentrations. It can be observed that all biocomposites exhibited an apparent porosity ranging from 0.10 to 0.16%.

When analyzing the porosity of the samples, it is noteworthy that the apparent porosity of the C3 biocomposite was higher than that of the C1 and C2 biocomposites. An increase in apparent porosity was observed for each of the biocomposites obtained with the incorporation of HAp. This behavior was expected due to the increased amount of HAp in PEEK. This phenomenon was also reported by Li et al. [53], who studied PEEK-based biocomposites for orthopedics. HAp, when processed at high concentrations, can hinder the complete sintering of grains. This occurs because the material’s density may limit the diffusion of calcium and phosphate ions between particles. Sintering can result in a network of interconnected microchannels and pores, which increase the material’s porosity. HAp granules may agglomerate during the molding process, creating voids between them. These voids remain as pores after sintering, contributing to the total porosity of the material.

According to Bastan et al. [54], the PEEK particles spread over the substrate and acted as a continuous matrix due to the applied heating temperature (above the melting point). The PEEK matrix can maintain mechanical integrity while the HAp particles provide in vitro bioactivity to the composite coating. Previous studies have shown that porous surfaces can provide a larger surface area and enhance blood supply and oxygen delivery within bone tissues [55,56,57,58]. Additionally, HAp can promote bone cell growth and regeneration, improve adhesion between bone tissue and the implant, and regulate the rate and direction of bone remodeling [53,58]. 

Understanding the density and porosity of HAp/PEEK biocomposites is extremely important because the more significant the chemical, physical, and structural similarity between the prosthesis and bone tissue, the lower the likelihood of future issues with the implant. The porous surface is essential in a bone repair biomaterial, as it aids in new bone formation and neovascularization, produces more excellent wettability, and promotes the diffusion of biological fluids. This facilitates adherence and enhances cellular activity, characteristics that are essential in grafts. According to Dorozhkin [33], porous structures increase the surface area, improving space for cell attachment and favoring chemical bonding with the adjacent tissue. Additionally, porosity regulates bioactivity as it directly affects structural permeability, which controls the initial rate of tissue regeneration and influences mechanical properties.

**Figure 6:**
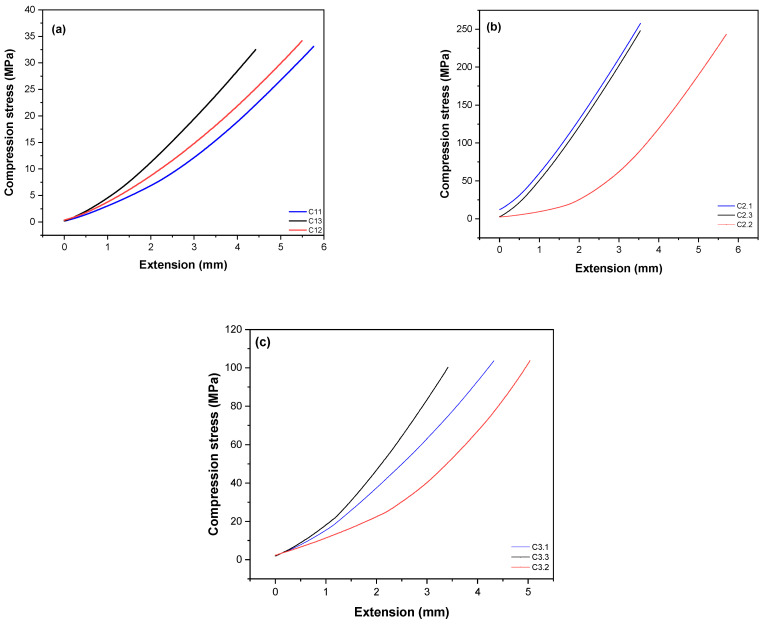
Compression strength curves as a function of extension for the biocomposites: (**a**) C1, (**b**) C2, and (**c**) C3.

Properly combining raw materials and processing techniques makes it possible to obtain biocomposites with high structural uniformity and mechanical strength values that make them suitable for various applications, including bone repair. Figure 6 illustrates the compressive stress vs deformation curves of the HAp/PEEK biocomposites with different HAp concentrations.

PEEK and hydroxyapatite-based biocomposites have shown characteristic behavior of semicrystalline materials in their compression strength curves, as observed in Figure 6. Despite its macromolecular rigidity, PEEK exhibits considerable deformation and can withstand significant levels of plastic deformation in both stress and compression. These materials are influenced by parameters such as temperature, test speed, and crystallinity, which can affect the penetration of HAp particles and consequently influence the morphology of the biocomposites, impacting their mechanical properties.

The results of the compression tests indicate that the mechanical strength of the biocomposites does not follow a linear response as the concentration of HAp increases in PEEK. For example, the C2 biocomposite exhibited an average compressive strength of 246 MPa ± 0.9, significantly higher than the other samples (C1: 34 MPa ± 0.8 and C3: 96 MPa ± 0.7). This behavior can be attributed to the uniform and well-dispersed distribution of HAp within the PEEK matrix, as previously observed in the EDS results. This confirmed the homogeneity of the constituent mixture in the C2 sample, resulting in higher compressive strength. On the other hand, SEM revealed areas of porosity and HAp particle aggregation in the C3 biocomposite. These defects act as weak points under load, leading to a decline in mechanical properties. In a study conducted by Zheng et al., it was demonstrated that an excess of HAp reduced the mechanical properties of the HAp/PEEK composite [58].

The results suggest that a well-refined and homogeneous microstructure, with good interfacial adhesion between PEEK and HAp, is essential for achieving a composite with excellent mechanical performance. The C2 biocomposite exhibited higher mechanical strength, which is attractive for biomaterial applications requiring compressive stress. According to Silva [59], materials for bone repair should demonstrate the strength to withstand mechanical forces while regenerating. However, this strength does not necessarily need to be equivalent to that of bone, as over time, there is a significant increase in compression, ranging from 10 to 30 MPa, due to the growth of bone tissue in vivo.

The agar diffusion test was chosen to analyze the cytotoxicity of the HAp/PEEK biocomposites. This method involves evaluating the effects of the samples through an agar layer, which protects the cells from mechanical damage and allows the diffusion of substances from polymeric samples. The discoloration index was obtained by averaging the halo measurements in four sample quadrants, starting from its edge, as presented in Table 2. The positive control had an index of three; the average discoloration zone was 0.88 cm, with a moderate cytotoxicity level. The negative control, as expected, did not cause cell lysis and was classified as non-cytotoxic. The HAp/PEEK sample tested by the agar diffusion method did not show halo formation, a result similar to the negative control. This result indicates the absence of cytotoxicity, as shown in Table 2.

**Figure 7:**
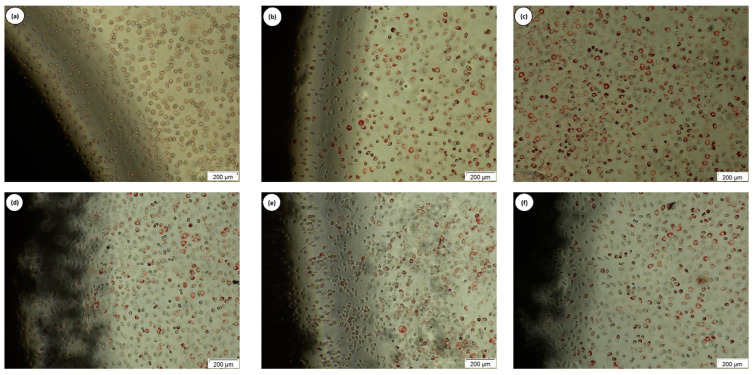
Images obtained by inverted digital microscopy at 100× magnification of the L929 cell line in the discoloration zones around the samples in the agar diffusion test: (**a**) positive control, (**b**) negative control, (**c**) blank, (**d**) C1, (**e**) C2, and (**f**) C3.

The images obtained from inverted digital microscopy were recorded after scanning the wells, prioritizing the sample’s contour to verify the occurrence of cell lysis. The images presented in Figure 7 were qualitatively evaluated for the presence or absence of cell lysis, and the results were compared with the positive and negative controls. The positive control (Figure 7a) caused complete clearing, clearly reflecting cell lysis under and around the sample, confirming the cytotoxic nature indicated in Table 2. On the other hand, the negative and white controls (Figure 7b,c), as well as the samples (HAp/PEEK) (Figure 7d–f), exhibited a vital fibroblastic cell layer, where the uptake of neutral red dye occurred after 24 h of incubation. This indicates the absence of cytotoxicity in the tested (HAp/PEEK) samples and their viability for biological use in general biomaterial applications. The blank well (Figure 7c) should be treated as a parameter to observe the uptake of neutral red dye by living cells.

These results indicate that the HAp/PEEK samples were not toxic to the cells, making them viable for biomaterial applications, especially in regenerative medicine. These findings are consistent with reports in the literature that evaluated the cytotoxicity of HAp/PEEK [60]. Therefore, these biocomposites have promising potential for biomedical applications, considering their biocompatibility and safety of the tested cells.

The HAp/PEEK (C2—40/60%) biocomposite showed better mechanical behavior, which favors its application as a high-performance biomaterial. Therefore, it is essential to consider that exposed orthopedic wounds are highly susceptible to bacterial infection, the primary cause of implant surgery failure. Thus, implants with antimicrobial activity can help prevent infections at the site [61]. Given this, the antimicrobial activity of the HAp/PEEK (C2—40/60%) biocomposite was evaluated, and the results are presented in Table 3. According to the results, the biocomposite C2 demonstrated antibacterial activity, with minimum inhibitory concentration (MIC) against *S. aureus*, *P. aeruginosa*, and *E. coli*. The bactericidal effect of the biocomposite C2 was observed at twice the MIC concentration. It is important to note that isolated PEEK has low antimicrobial properties, which limits its application [62]. Thus, several strategies have been employed to enhance the antimicrobial activity of PEEK, such as surface modification or combining it with other materials [63]. In this regard, using coating materials like hydroxyapatite (HAp) is considered to improve the antibacterial properties of the implant material [64], as previously verified by the EDS results, which confirmed the homogeneity of the constituent mixture in this biocomposite.

The study conducted by Wang et al. [65] demonstrated that bacterial biofilms formed and reached a thickness of about 90 µm with live bacteria visible on the surface of isolated PEEK. In contrast, bacterial cells were killed on the surface of the PEEK/nanofluorohydroxyapatite biocomposite, suggesting the antimicrobial activity of this composite. This bioceramic material affects both Gram-positive and Gram-negative bacteria due to its ability to penetrate bacterial cell walls through electrostatic interaction with the cell wall [66]. Additionally, other studies have shown activity through the production of reactive oxygen species (OH^−^, H_2_O_2_ e O_2_^−2^) that can disrupt bacterial membranes, abrasive surface ordering due to surface effects, and aggregates that may contribute to mechanical damage to the bacterial cell membrane [67].

## 4. Conclusions

The synthesis of hydroxyapatite (HAp) and polyether ether ketone (PEEK) biocomposites using high-energy ball milling has proven to be a promising approach for creating materials for bone regeneration. The incorporation of HAp into PEEK resulted in significant changes in the material’s morphology, particularly regarding porosity and mechanical strength. Among the analyzed biocomposites, C2, with 40% HAp, stood out for its effective antibacterial activity against *S. aureus*, *P. aeruginosa*, and *E. coli*, showing notable minimum inhibitory concentration (MIC) and bactericidal effect at concentrations above the MIC. Additionally, cytotoxicity tests revealed that C2 is non-toxic to cells, confirming its viability in biomaterials and safety in contact with biological tissues. The developed system offers significant potential for various medical applications, especially in bone restoration, due to its well-balanced combination of mechanical and biological properties.

## Figures and Tables

**Table 1:** Degree of cytotoxicity.

Degree	Cytotoxicity	Description of the Cytotoxicity Zone
0	Absence	Absence of bleaching around or under the sample.
1	Light	Bleaching zone limited to the area under the sample
2	Soft	Sample bleaching zone size less than 0.45 cm.
3	Moderate	Sample bleaching zone size less than 0.45 cm to 1.0 cm.
4	Severe	Size of the sample bleaching zone greater than 1.0 cm, but not involving the entire plate.

**Table 2:** Results of the agar diffusion test on HAp/PEEK biocomposites.

Tested Material	Degree Discoloration	Cell Lysis	Interpretation
Positive control	3	4	Moderate cytotoxicity
Negative control	0	0	Non-cytotoxic
white	0	0	Non-cytotoxic
C1	0	0	Non-cytotoxic
C2	0	0	Non-cytotoxic
C3	0	0	Non-cytotoxic

**Table 3:** MIC/MBC of Biocomposite C2.

Microrganisms	MIC/MBC (µg.mL^−1^)			
	C2	CIPRO	OXA	CFT
*S. aureus* ATCC (25923)	1562.5/3125	200	200	-
*P. aeruginosa* ATCC (27853)	1562.5/3125	200	-	200
*E. coli* ATCC (25922)	390.62/781.25	200	-	200

ATCC—American Type Culture Collection; MIC—Minimum Inhibitory Concentration; MBC—Minimum Bactericidal Concentration; CIPRO—Ciprofloxacin Hydrochloride; OXA—Oxacillin; CFT—Ceftazidime.

## Data Availability

The original contributions presented in the study are included in the article; further inquiries can be directed to the corresponding author.
